# The effect of zinc sulfate on immunologic response to recombinant hepatitis B vaccine in elderly: Zinc sulfate and immunologic response to recombinant hepatitis B vaccine

**Published:** 2011-01-01

**Authors:** Mandana Afsharian, Siavash Vaziri, Ali Reza Janbakhsh, Babak Sayad, Feizollah Mansouri, Javad Nourbakhsh, Keyghobad Qadiri, Farid Najafi, Maria Shirvanii

**Affiliations:** 1 Kermanshah Liver Disease and Hepatitis Research Center, Kermanshah University of Medical Sciences, Kermanshah, IR Iran; 2 Kermanshah Infectious Disease Research Center, Kermanshah University of Medical Sciences, Kermanshah, IR Iran; 3 Kermanshah Health Research Center (KHRC), Kermanshah University of Medical Sciences, Kermanshah, IR Iran

**Keywords:** Hepatitis B, Vaccination, Zinc sulfate, Immunity

## Abstract

**Background:**

Hepatitis B is the major cause of chronic hepatitis and cirrhosis in Iran. Sanitation and immunization is one of the most effective measures for prevention of the disease which is now widely used in developing countries. However, the immune response to the vaccine varies by age.

**Objectives:**

To determine the effect of zinc sulfate on immune response to hepatitis-B vaccine in elderly.

**Patients and Methods:**

In a clinical trial on 140 subjects aged ?40 years with a body mass index (BMI) <30 kg/m2, and without any co-morbid disease were recruited. Those who had negative hepatitis B core antibody (102 persons) were randomly allocated to two groups. The trial group received hepatitis B vaccine plus 200 mg zinc sulfate daily for 30 days and the control group received vaccine plus placebo.

**Results:**

52 of 102 people were female (51%). The two studied groups were comparable in terms of age, gender, and smoking habits. The mean antibody production in the intervention and control groups was 116.93 and 157.37 mIU/mL, respectively (p=0.22). No statistical differences were observed between the two groups in terms of proportion of people who were protected after vaccination (26.0% and 36.5% in people with and without zinc, respectively).

**Conclusions:**

This study revealed that zinc sulfate has no effect in level of immunity among elderly.

## Background

Hepatitis B is a major public health problem with two billion people infected worldwide [1]. HBV is the most important cause of viral hepatitis in the world that can cause cirrhosis and liver cancer. More than 350 million people are chronic carriers of HBV and approximately 75% of them live in Asia and Africa [2]. Two million people die of the disease annually in the world [3]. The major routes of HBV transmission are via blood transfusion and hemodialysis, dental surgery, tattooing and drug abuse (IDU). About 1.5 million people in Iran are living with HBV infection [2]. The prevalence of infection in Iran is estimated to be 2.14% in men and 2.55% in women. However, the prevalence of people with positive HBsAg varies between different provinces: Golestan (6.3%), Tehran (2.2%), East Azerbaijan (1.3%), Hamadan (2.3%), Isfahan (1.3%), Kermanshah province (1.3%) and Hormozgan (2.4%) [4]. It has been reported that the prevalence of people with positive HbsAg in Iran decreased from 2003 to 2005 [5]. Vaccination is one of the most effective way of preventing disease, decreasing disability, and death from all infectious diseases [6]. It seems that in endemic regions hepatitis B vaccination have a significant effect on reducing the chronic infection and carrier state, but may not reduce the infection rate [7]. However, the immune response to the vaccine is age dependent. Different studies as well as meta-analyses have shown that immune response to vaccines decreases by increasing age. This is why hospital admission rate has been increasing in elderly people. According to such studies between10% and 50% of the old age people cannot be protected even after vaccination [6]. In order to improve the immunogenicity of vaccination, different methods such as changes in vaccination method and adding supplements such as, levamisole, cimetidine, zinc sulfate and some other drugs have been investigated [8].

## Objectives

This study was conducted to evaluate the effect of zinc sulfate as an adjuvant to increase the level of antibody production after hepatitis B vaccination in elderly.

## Patients and Methods

In a clinical trial conducted at Imam Reza Hospital, the main referral center in western Iran in Kermanshah province, from 140 subjects over 40 years old, 102 that were negative for HBcAb (measured by competitive ELISA, Biomerieux, USA), enrolled into this study and randomly allocated into two intervention and control groups. Subjects were given detailed information about the study and written informed consent was obtained. A questionnaire including information regarding demographic factors, history of exposure to hepatitis B, any previous allergy, history of pervious hepatitis B vaccination was completed by trained interviewers. The intervention group was given hepatitis B vaccine (Hepavax, Korea) plus 200 mg zinc sulfate capsule (Alhavi, Iran); the control group was given hepatitis B vaccine (Hepavax, Korea) plus placebo (capsule filled by sugar). Three vaccination injections were given intra-muscularly using rapid method on the first day, and the next 10 days following, and finally in 22 days after the first injection [9] Anti-HBs antibody was measured by competitive ELISA (Biomerieux, USA) at the same hospital lab one month after the last vaccine injection in both groups. For the purpose of this study with assumption of 60% and 90% of people who reached to the protective level in control and trial group, we calculated a sample size of 102 people for the study to be able to reject the null hypothesis that the proportion for trial and control groups are equal with a study power of 0.95. Type I error probability was set to 0.05. Uncorrected x(2) test was used to test the null hypothesis. Statistical analyses were performed using Stata ver 8. A p < 0.05 was considered statistically significant. Mean serum antibody between two groups was compared by Student's t test. To compare the number of people who were immune after vaccination in two groups we used x(2) test. Using univariate and multivariate logistic regression, we investigated the effect of different factors on seroconversion.

## Results

**Figure 1:**
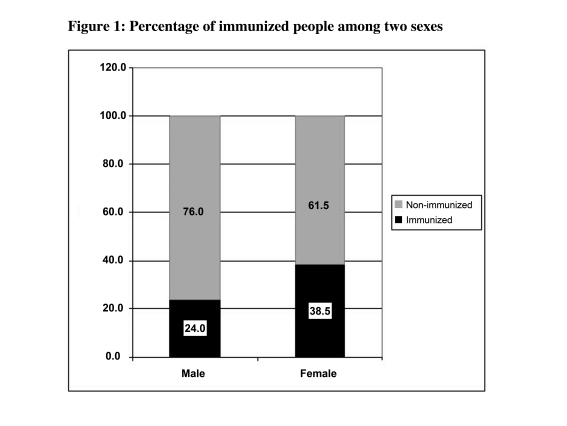
Percentage of immunized people among two sexes

**Figure 2:**
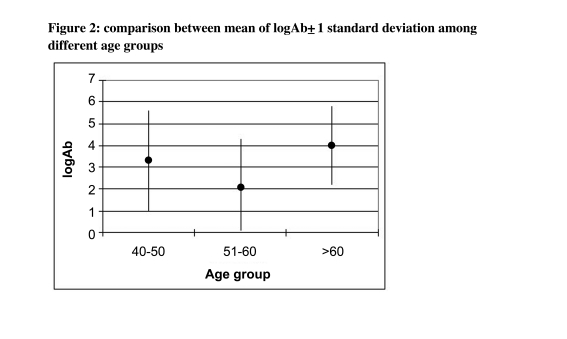
Percentage of immunized people among two sexes

**Figure 3:**
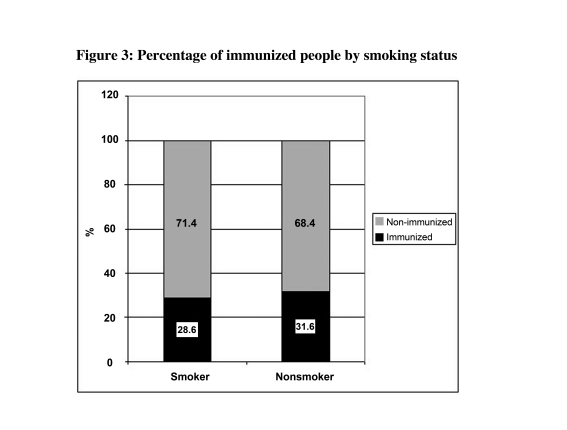
Percentage of immunized people by smoking status

The subject of this study included 52 women (51.0%) and 50 men (49.0%). Out of the 102 participants, seven (6.9%) were cigarette smokers (4% in the intervention and 9.6% in the control group). From 102 participants, 50 in the trial group received zinc sulfate with three injections of the vaccine. The median age of participants was 47 (range: 40-73) years. There were no statistical significant differences between the intervention and control groups in terms of main confounding and background variables such as gender, age and smoking habits and body mass index (BMI) (all p>0.1) (Table 1). Comparing mean antibody level and the proportion of people who reached the protective level, both groups were similar (Table 1). While the distribution of male and female participants among trial and control groups was similar (Table 1), in both groups slightly greater proportion of females reached the protective level (Figure 1). Similar findings were observed when we compared the mean of natural logarithm of antibody level in two sexes in both groups (3.2±2.3 and 3.3±2.0 among males in the control and intervention groups, respectively, and 3.8±2.4 and 3.1±2.3 among females in the control and trial groups, respectively). While the proportion of smokers among two groups was similar (Table 1), smoking has no significant effects on the proportion of immunized people after three vaccine injections (Table 2). None of smokers in the trial group developed the protective antibody level (Figure 2). The mean age among the studied groups was also similar (Table 1). Although non-statistically significantly, the proportion of protected people decreased by growing age in both univariate and multivariable analyses (Table 2). Figure 3 shows the mean of log Ab level in both groups by age. Table 2 shows the result of univaritate and multivariate analyses of all variables including "group" on final immunity after three vaccine injections. After adjustment, none of the studied variables had effect on immunity.

**Table 1:** Comparison of characteristics and Ab level among the two groups

**Variable**	**With Zinc **(n=50)	**Without Zink**(n=52)	**p-value**
**Gender** (Female %)	50	51.9	0.85
**Age **(mean±SD, year)	49.1 ± 8.0	48.4 ± 8.3	0.67
**Smoking **(%)	4.0	9.6	0.26
**BMI**	25.8 ± 2.0	25.2 ± 2.3	0.14
**Ab level **(mIU/mL)	116.93	157.37	0.22
**Immunity **(%)	26.0	36.5	0.25

**Table 2:** Effect of different factors on immunity after hepatitis B vaccination using logistic regression analysis

**Variable**	**Univariate analysis** (95% CI)	**Multivariate analysis **(95% CI)
**Gender** (Female)	1.9 (0.84-4.7)	1.68 (0.66-4.26)
**Age**	0.95 (0.89-1.0)	0.96 (0.91-1.0)
**Smoking**	0.87 (0.16-4.7)	0.95 (0.19-5.9)
**BMI**	0.92 (0.76-1.11)	0.91 (0.74-1.12)
**Group **(trial)	0.61 (0.26-1.42)	0.64 (0.26-1.56)

## Discussion

Zinc is an essential micronutrient for human metabolism that catalyzes more than 100 enzymes, facilitates protein folding, and helps to regulate gene expression. Patients with malnutrition, alcoholism, inflammatory bowel disease, and malabsorption syndromes are at increased risk of zinc deficiency. Symptoms of zinc deficiency are nonspecific, including growth retardation, diarrhea, alopecia, glossitis, nail dystrophy, decreased immunity, and hypogonadism in males. In developing countries, zinc supplementation may be effective for the prevention of upper respiratory infection and diarrhea, and as an adjunct treatment for diarrhea in malnourished children. However, current data do not support the positive effect of zinc supplementation on, wound healing, or human immunodeficiency virus. Zinc is well-tolerated at recommended dosages. However, adverse effects of long-term and high-dose zinc consumption are: effect on decrease in immunity and high-density lipoprotein cholesterol levels, anemia, copper deficiency, and possible genitourinary complications [10]. All kinds of immune cells show decreased function after zinc depletion. In such situation, all functions related to monocytes and T cells are impaired, in natural killer cells and neutrophils cytotoxicity activity and phagocytosis is reduced, respectively. However, auto-reactivity and all reactivity will increase [11]. In fact, enhancing the immune response to the vaccination after hepatitis B among older peoples is one of the most important issues. There are large number of studies investigating the different methods for increasing the immune response after vaccination such as use of different adjuvants (such as levamisole, cimetedine and zinc sulfate), different methods of vaccination (whether intradermal or intramuscular as well as rapid or conventional methods). For example, vaccination via the intradermal route (ID) is considered as a method that may be more effective than the conventional intramuscular (IM) route [12]. However, such general belief is not supported by findings from all studies [13]. While other factors such as smoking status, BMI, and age may have effect on Ab production after vaccination, effect of different adjuvants have been investigated in different studies. It is generally believed that addition of levamisole to hepatitis B vaccine can enhance the immunological response [12]. Regarding the use of zinc, while there are some controversies on the effect of zinc supplementation on immune status, it has been suggested that daily use of up to 40 mg zinc has no adverse effects on immune status of healthy people [14]. However, different studies investigating the effect of zinc on proportion of seroconversion showed no effect [15][16]. One study about the effect of zinc sulfate on increasing antibody response in hemodialysis patients was done in Italy in 1990. This study showed 50% increase in antibody production in non-responders (mean antibody production was 124.6 mIU/mL) [15]. Another study on the effect of zinc sulfate on increasing antibody response in hemodialysis patients was done in Holland in 1991. In that study the patients was categorized into two groups (n=7, each group). In one group, hepatitis B vaccine with zinc sulfate was given and in another group hepatitis B vaccine without zinc sulfate was administered. Results showed no difference in antibody production between the two studied groups [16]. Another study on the effect of zinc sulfate on increasing antibody response in elderly people was done in Ireland in 2007. In this study, one group received zinc sulfate capsules, 15 mg daily for six months; the other group was given 40 mg zinc sulfate daily for six months. Result showed no difference in the antibody production between the two study groups [14]. According to the results of some studies that discussed previously we thought that zinc might stimulate antibody response in the non-responders to hepatitis B vaccine. One of the most important of non-responder groups are old people. This study showed no statistical difference between the two study groups in terms of gender and weight in antibody production. Random allocation of people into two groups created a similar distribution of all background and confounding variables between two studied groups. After adjustment for all other variables, there was no effect for smoking, group and sex on seroconversion. It is worth mentioning that for the purpose of this study we used accelerated method for vaccination and that may contribute to our final results regarding no effect in the seroconversion of people allocated to two groups. Accelerated HB vaccination can shorten duration of immunization we performed this clinical trial for showing its effectiveness [9]. Future studies should aim at investigating the effect of zinc sulfate on seroconversion after a simple method vaccination (0, 1 m, 6 m). On the other hand, future studies need to have a larger sample size to find out possible effects of zinc as well as other variables such as age, gender, smoking and BMI, since in the current study, it is difficult to explain the independent effects of such variables.
